# The associations of childhood and adolescence psychosocial risks with social relations in midlife

**DOI:** 10.1038/s41598-026-58873-5

**Published:** 2026-07-01

**Authors:** Marko Elovainio, Kaisla Komulainen, Katja Pahkala, Suvi Rovio, Terho Lehtimäki, Jorma Viikari, Ilmari Määttänen, Olli T. Raitakari, Laura Pulkki -Råback

**Affiliations:** 1 Research program unit, Faculty of Medicine, Department of Psychology, University of Helsinki, P.O.Box 63, 00014 Helsinki, Finland; 2 Faculty of Medicine, Department of Psychology, University of Helsinki, Helsinki, P.O.Box 63, 00014 Finland; 3 Finnish Institute for Health and Welfare, Mannerheimintie 166, Helsinki, 00300 Finland; 4 Centre for Population Health Research, University of Turku and Turku University Hospital, Kiinamyllynkatu 10, Turku, 20520 Finland; 5 Research Centre of Applied and Preventive Cardiovascular Medicine, University of Turku, Kiinamyllynkatu 10, Turku, 20520 Finland; 6 Paavo Nurmi Centre and Unit for Health & Physical Activity, University of Turku, Turku, Finland; 7 Department of Clinical Chemistry, Fimlab Laboratories, and Finnish Cardiovascular Research Center - Tampere, Faculty of Medicine and Health Technology, Tampere University, Tampere, Finland; 8 Department of Medicine, Division of Medicine, University of Turku, Turku University Hospital, Turku, Finland; 9 Department of Clinical Physiology and Nuclear Medicine, Turku University Hospital, Turku, Finland; 10 InFLAMES Research Flagship, University of Turku, Turku, Finland; 11 Faculty of Medicine, University of Helsinki, Haartmaninkatu 3, Helsingin yliopisto, FI-00014 Finland

**Keywords:** Loneliness, Social support, Social network, Early risk factors, Young Finns Study, Risk factors, Psychology and behaviour

## Abstract

We examined whether the accumulation of psychosocial risk factors (socioeconomic risks, health behavioural risks, stressful life-events and psycho-emotional risks) in childhood and adolescence contributes to insufficient social relations (loneliness, frequency and heterogeneity of social contacts and social support) in adulthood participants from the longitudinal Young Finns Study with a 38-year follow-up (*N* = 1787, 56% female). Results from regression models indicated that a higher cumulative number of risks in childhood and adolescence was associated (1-SD increase) with higher loneliness (B = 0.32, 95% CI 0.19 ; 0.45), lower perceived social support (B=-0.32, 95% CI -0.45 ; -0.19), lower frequency of social contacts (B = 0.19, 0.06 ; 0.32), and lower heterogeneity in social contacts (B=-0.33, -0.47 ; -0.20) in midlife. Causal mediation analysis suggested that depressive symptoms partially mediate the relationship between cumulative childhood and adolescent psychosocial risks and adult social relations (mediation proportion range between 7% and 18%). Educational attainment mediated the relationship between cumulative psychosocial risk and social relations heterogeneity by 8%, while income mediated the association with loneliness, social support and social relations heterogeneity by 9% to 11%. In conclusion, this study underscores the potentially profound and lasting impact of psychosocial risks encountered during childhood and adolescence on social relations in adulthood.

**Supplementary Information:**

The online version contains supplementary material available at https://doi.org/10.1038/s41598-026-58873-5.

## Introduction

Childhood and adolescence are critical periods during which family-related psychosocial environments can exert profound and enduring effects on individuals’ health and wellbeing^1–4^.

These family risk factors include low parental socioeconomic status^5^, parental mental health problems^6^, parental alcohol use or antisocial behaviour^7^, an unhealthy diet^8^, a poor quality of emotional environment (such as maltreatment)^9^, and exposure to stressful life events (such as parental divorce)^1,10^. Studies suggest that the risk of mental or physical health problems is particularly high when multiple risk factors accumulate^1,3,11^.

There is also growing evidence that psychosocial risks experienced in childhood are associated with negative social outcomes later in life, including a higher risk for loneliness, social isolation, and lower social support^12,13^. These outcomes are not only distressing in themselves but have also been linked to elevated risks for mental and physical health problems, such as depression^14,15^, cardiovascular disease and type 2 diabetes^16,17^, serious infections^18^ and even excess mortality^19^. While most existing studies have focused on the associations between individual childhood risk factors and adult social outcomes, cumulative exposure to multiple psychosocial risks during childhood and adolescence may carry a higher risk for adverse social outcomes than any single risk factor alone^20–22^.

The accumulation of risk factors can be conceptualized within the ‘risky family’ model^5^, which characterizes risky family environments as marked by aggression, conflict, and unsupportive relationships. Within this model, specific family characteristics – such as low family socioeconomic status (SES) and stressful life events – contribute to an increased likelihood of conflict and unsupportive relationships within the family. Additionally, parental health behaviours – such as alcohol use or smoking – shape the family psychosocial climate and serve as maladaptive behavioural models for children. Together, by fostering conflict and negative relationships, a risky family environment may have a lasting impact on child’s future social relationships.

In addition, other theoretical frameworks, such as attachment theory and evolutionary theory^23^, offer valuable insights into how early adversity and associated disruptions in parent-child relationships can lead to later difficulties in forming and maintaining social relationships. Attachment theory posits that emotional maltreatment can disrupt the formation of secure attachments by negatively shaping perceptions of others and fostering a worldview in which others are perceived as untrustworthy and even threatening^24^. Similarly, the evolutionary model of loneliness suggests that humans are motivated to seek social connection as it confers a sense of safety^25^. Early exposure to a risky psychosocial environment may erode this sense of safety, leading to the perception that one’s social connections are deficient and not meeting essential emotional and relational needs.

Several mechanisms may explain how early psychosocial risks contribute to later social difficulties. As proposed by theoretical models, a risky family environment can disrupt the formation of secure attachments, which in turn can contribute to internalizing symptoms such as depressiveness^26^. Depressive symptoms can, in turn, hinder the ability to form and maintain social relationships^27^. Additionally, chronic stress during early life has been shown to increase emotional reactivity and sensitivity to perceived threats^28^. Individuals may attempt to regulate these negative emotions by adopting maladaptive coping strategies, including smoking, overeating, or excessive alcohol use^28^. Finally, a risky family environment has been associated with lower educational achievement^29^, indicating that socioeconomic factors may be one pathway linking early-life adversity to impaired social functioning later in life.

In this longitudinal study, we examined whether the accumulation of psychosocial risk factors measured in youth in 1980 is associated with loneliness, low perceived social support, low frequency of social contact, and social network heterogeneity in later mid-life measured 38 years after baseline in 2018. The early-life psychosocial factors included family characteristics related to socioeconomic disadvantage, emotional adversity, parental health behaviours, and exposure to stressful life events, together forming a cumulative measure of psychosocial factors in youth. We also examined whether depressive symptoms^30^, socioeconomic status^31^ or poor health behaviours^32,33^ in 2011 mediate the associations between early psychosocial risks and mid-life social relations. This study extends previous studies by assessing the longitudinal associations of childhood psychosocial risks that had not previously been examined in relation to domains of social relations in late adulthood. In our study, the childhood factors were measured prospectively in childhood, which eliminates the recall bias typically present in studies using retrospective designs. We hypothesized that the accumulation of risk scores across different domains present from age 3 to 18, is associated with higher risk of negative sociel relations domains, including loneliness, poor social support, low network hererogeneity and low frequency of social contacts.

Bottom of Form.

## Results

**Table **1, presents the characteristics of the sample (*N* = 1,787, 57% female). In childhood or adolescence, 21.5% of the participants had experienced cumulative psychosocial risks. Among the specific domains, psycho-emotional risks were the most common, reported by 32.9% of participants.

**Fig. 1:**
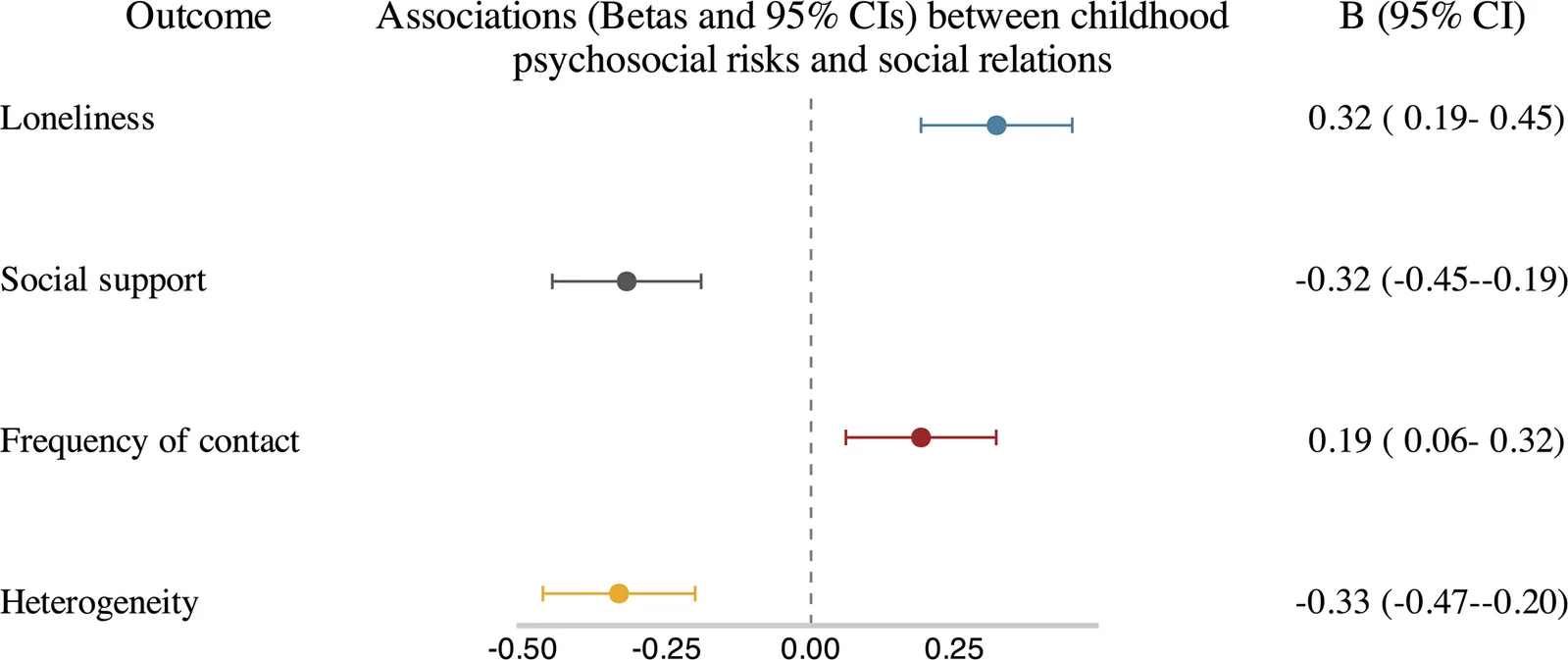
Association (1-SD increase) between cumulative childhood/adolescence psychosocial risk domains and four indicators of social relations in adulthood (linear regression analyses), adjusted for age and sex.

Results from regression models evaluating the exposure-outcome associations are presented in Fig. 1. A higher number of cumulative risks in childhood and adolescence was associated (1-SD increase) with higher loneliness (B = 0.32, 95% CI 0.19; 0.45), lower perceived social support (B=−0.32, 95% CI −0.45; −0.19), lower frequency of social contacts (B = 0.19, 95% CI 0.06; 0.32) and lower social contacts heterogeneity (B=−0.33, 95% CI −0.47; −0.20) in adulthood (Fig. 1). These associations were largely replicated when focusing on the specific risk domains (**Supplement Fig. 2**).

Table 1,2 shows the associations between cumulative childhood psychosocial risks and potential mediators. Cumulative childhood and adolescent risks were associated with greater odds of depressive symptoms (OR = 1.55, 95% CI 1.07; 2.21), smoking (OR = 2.33, 95% CI 1.57; 3.43), lower education (OR = 2.80, 95% CI 2.01; 3.90) and lower income (OR = 1.83, 95% CI 1.33; 2.51), but not with alcohol consumption or obesity. Of the potential mediators, depressive symptoms were associated with loneliness (B = 0.69, 95% CI 0.53; 0.84), lower social support (B = −0.52, 95% CI −0.67; −0.37) and lower social contacts heterogeneity (B = −0.30, 95% CI −0.46; −0.15) and low income with loneliness (B = 0.27, 95% CI 0.14; 0.41), lower social support (B = −0.29, 95% CI −0.42; −0.16), higher frequency of social contacts (B = 0.21, 95% CI 0.07; 0.34) and lower social contacts heterogeneity (B = −0.30, 95% CI −0.44; −0.6) (Table 2,3,4).

Results from the causal mediation analysis with adult depressive symptoms as a potential mediator between cumulative risks in childhood and social relations in late mid-life are presented in Table 3,4. The indirect effect of cumulative psychosocial risk on loneliness attributable to depressive symptoms was significant, but relatively small, with an estimated 18% of the association mediated. For social support and network heterogeneity the estimated proportions mediated by depressive symptoms were 17% and 7%, respectively, while depressive symptoms did not mediate the association between cumulative risks and frequency of social contacts. As shown in Table 4,5 none of the adult health behaviours (smoking, obesity, or alcohol consumption) acted as mediators in the relationships between childhood and adolescent cumulative psychosocial risk and any of the social relations outcomes.

Table 5,6 which presents the mediation results for socioeconomic factors, shows that low adult education acted as a mediator between cumulative childhood and adolescence psychosocial risks and social contact heterogeneity, with an estimated 8% of the association mediated. No indirect associations via education were observed for the other social outcomes. In contrast, low adult income emerged as a mediator in the associations of adverse childhood environment with loneliness, lower social support and lower social relations heterogeneity, with the estimated proportions mediated ranging from 9% to 11% (Table5,6).

## Discussion

This study provides prospective longitudinal evidence for the relationship between childhood and adolescent psychosocial risk factors and multiple indicators of social relations in mid-life. Using a prospective Finnish cohort followed up for 38 years, we observed that the accumulation of psychosocial risks in childhood and adolescence was robustly associated with higher loneliness, lower perceived social support, less frequent social contact and lower social relations heterogeneity in midlife. These associations were partially mediated by adult depressive symptoms and socioeconomic disadvantage, suggesting that early psychosocial adversity may shape later social outcomes through both psychological and material pathways.

There is a growing body of evidence linking early-life environments to later life outcomes. Childhood and infant attributes, such as socio-economic status^34^, psychological problems^35^, and physical health^36^, have all been linked to a variety of health and wellbeing outcomes across the life course. Moreover, the accumulation of adversities in childhood has been shown to predict multiple indicators of adult wellbeing, including depressive symptoms, cardiovascular health and obesity^1,32,37,38^. Despite the recognized importance of childhood experiences, few empirical studies have assessed the long-term prospective associations between psychosocial risks in early life and adult social relationships measured decades later. The findings of this study help to address this gap in the literature by providing prospective evidence that early psychosocial environments have long-term associations with adult social relationships. The consistent and robust associations observed between adverse psychosocial environment in childhood or adolescence and various indicators of social relations in midlife suggest that early life experiences may be critically important for the formation and maintenance of adult social relations. Our study adds to existing evidence that early psychosocial risks may have enduring effects that extend well into adult life, influencing the quality and quantity of social interactions, loneliness, and perceived social support.

Our results highlight that loneliness – a significant public health concern due to its strong associations with various mental and physical health problems^16,31,39^– may be rooted in early-life experiences. This finding is consistent with an earlier study suggesting intergenerational continuity in loneliness^40^, although longitudinal studies remain scarce. Our findings also align with theoretical frameworks suggesting that adverse childhood experiences may contribute to social withdrawal and difficulties in forming and maintaining relationships later in life, which may manifest as loneliness.

In addition, we found that early psychosocial risks were associated with lower perceived social support, less frequent social contact, and lower social network heterogeneity in adulthood. These findings suggest that early adversity may limit not only the number of social contacts but also their *diversity*. This lower heterogeneity could reflect a constrained social environment or a preference for familiar and emotionally safer relationships, consistent with predictions from attachment and evolutionary theories^23^. A lack of diverse social interactions may limit opportunities to turn to different people for different needs or situations, making the individual more reliant on a small number of contacts and potentially heightening vulnerability to disruptions in these relationships.

Multiple mechanisms have been proposed to explain how early psychosocial risks influence social relations in adulthood. In this study, we identified adult depressive symptoms and socioeconomic status as partial mediators of the relationship between early psychosocial risks and adult social relations. This mediation suggests that the pathway from early adversities to adult social outcomes may be explained, at least in part, by the psychological and economic consequences that follow from adverse early life experiences^40^. Depressive symptoms, often rooted in early adversities^1^, can impair social functioning by reducing both the motivation to engage with others and the ability to maintain social relationships. Similarly, socioeconomic disadvantage may be a consequence of early psychosocial risks, limiting access to social opportunities and resources needed for successful social integration in adulthood^41^.

It is noteworthy that health behaviours did not emerge as significant mediators in the association between childhood and adolescent psychosocial risks and negative social outcomes in adulthood. This finding was somewhat unexpected, given prior evidence showing that behaviours such as smoking or excessive alcohol use have intergenerational continuity^42^ and have been linked to problems in social relationships^43^. One possible explanation for this null finding is that the associations partly reflect shared underlying risk factors – such as early adversity – rather than direct causal pathways from health behaviours to social difficulties.

One of the key strengths of this study is the prospective data from a general population sample, spanning across 40 years, which allowed us to explore the long-term associations between childhood exposures and adult social relationships. Social relationships were assessed using four different indicators that explore both structural and functional aspects of social interaction and vary in their levels of subjectivity^1^. Treating measured outcomes as continuous data avoids information loss and maintains statistical power, can bias estimates, and complicates interpretation—especially since modern methods like linear regression handle the full variability effectively^44^.Additionally, the robustness of the findings across various social relation indicators lends further credibility to the observed associations. A further strength lies in the temporal structure of our causal mediation models. Owing to the longitudinal design, the mediators were assessed after the exposure but before the outcomes, which is methodologically optimal for evaluating mediation. The cumulative psychosocial score was based on binary variables. Combining various distinct risk factor into a single cumulative risk score treats all risks as having an equal impact on potential outcomes that is not always well justified. Cumulative binary scores have, however, the advantage of being parsimonious, they make no assumptions about risk factor collinearity, and they enable the testing of additive effects over a range of exposures^22^. It has been shown that these psychosocial factor domains only moderately accumulate, but, when they do, such an accumulation of negative factors may be particularly strongly associated with later social and health outcomes^45^.

Despite the strengths of this study, several limitations must be acknowledged. First, although the mediation models were temporally well-structured, the observational nature of the study precludes causal inferences. Although robust associations were observed, it is possible that unmeasured or residual confounding factors may have influenced the results. For instance, shared parental and individual genetic predispositions may impact several variables simultaneously. Additionally, although the original sample was randomly selected from the general population, the subset of participants with complete longitudinal data may not be representative of all demographic groups, limiting the generalizability of the findings. In addition, selective attrition may induce bias in the observed associations. The sample attrition in our study was relatively large and due to dropping out before the final study collection phase^46^. We acknowledge that attrition is higher among young men and individuals with lower socioeconomic position and although this may introduce some bias, we do not consider it a major threat to the validity of the study. Future research should aim to replicate these findings in diverse populations and explore potential mechanisms underlying the observed associations. The data used in this study were drawn exclusively from Finland, which may limit the generalizability of the findings. Finland is a high-income country with lower levels of income inequality compared to most other countries ^81^, and the Young Finns Study dataset, which is very homogeneous in terms of ethnicity, comprising white individuals of Finnish origin. These aspects may further limit the generalizability of our findings. Finland does not represent an extreme case within Europe with respect to key indicators of social relations. For example, population levels of loneliness in Finland fall near the middle of the distribution across European countries, according to a 2023 EU Joint Research Center survey (EU, 2023). This suggests that the results are unlikely to be substantially biased by the national context. Effect sizes were modest — a one-unit increase in cumulative risk score was associated with a 0.32 SD reduction in social support and similar increase in loneliness. While small by conventional benchmarks, a shift of this magnitude may carry practical relevance in social health research, where even moderate increases in loneliness or reductions in contact frequency have been linked to adverse outcomes.

To conclude, our findings underscore the importance of psychosocial risks encountered during childhood and adolescence for social relationships in adulthood. Our findings based on a cumulative risk exposure highlight that it is not only single adversities, but their accumulation, that may affect future social relations. While previous research – mostly relying on retrospective designs – has linked specific childhood adversities such abuse or neglect^12^ with increased loneliness in adulthood, our findings extend this knowledge by demonstrating prospective associations between multiple accumulated risk factors and a broad range of adult social outcomes.

Our study motivates further research on interventions aimed at reducing psychosocial risks in childhood and adolescence to support adult social relationships. Programs that enhance social skills, resilience, and coping mechanisms in young people facing adversities could mitigate the long-term negative effects on social relations. Equally important is addressing the root causes of risk accumulation during childhood, such as socioeconomic disadvantage, poverty, and parental stress, and ensuring adequate mental health support for families. Furthermore, addressing the mediating factors, such as offering mental health services to reduce depressive symptoms, could mitigate the consequences of early adversity for later social outcomes.

We showed that depressive symptoms and financial difficulties mediated the associations of early adversity with social problems later in life, but future research should explore a broader range of pathways linking early psychosocial risks to adult social relations. These may include, for instance, heightened sensitivity to threat, altered nervous system functioning, genetic and epigenetic predispositions, development of social skills, the ability to trust others, and cultural influences. Moreover, longitudinal studies involving diverse populations are essential to improve generalizability of these findings. Such research would also help clarify potential variation in how early-life experiences may shape social outcomes across different population groups. A deeper understanding about the mediators and mechanisms involved will be crucial for designing more effective intervention strategies for individuals with histories of early psychosocial adversities.

## Methods

### Sample

Data were from the ongoing prospective Young Finns Study (YFS) (http://youngfinnsstudy.utu.fi), which comprises six Finnish birth cohorts followed at several life stages from childhood to adulthood. This multidisciplinary study has been conducted in five university cities in Finland with medical schools (i.e., in Helsinki, Kuopio, Oulu, Tampere, and Turku) and the regions surrounding them^47^. The subjects were randomly selected from Finnish national registry among the chosen age groups and the five study districts. The YFS is conducted in compliance with the Helsinki Declaration and approved by the ethical committees of the Hospital District of Southwest Finland (Dnro 68/1801/2017 § 255). All individuals and/or legal guardians have signed a written informed consent to take part in the study.

The baseline study in 1980 included 3596 children and adolescents aged 3–18 years, representing six age cohorts (3, 6, 9, 12, 15, and 18 years). Participants were randomly selected from the Finnish national register, which covers the entire country and represents the general population. Indicators of social relations were assessed in the most recent follow-up in 2018 when the participants were on average 49 years old. We excluded all the participants that had missing values in any of the exposures measured in 1980 (*n* = 1012) or potential mediators/confounders measured in 2011 (*n* = 616) or outcome measures that were measured at the last data-collection step in 2018–2020 (*n* = 154). The final study sample was 1787 (56% female). For flow chart of study sample selection see **supplement** Fig. 1.

### Measures

#### Social relations

Social relations in midlife were assessed with four scales: loneliness, perceived social support, frequency of social contact, and social network heterogeneity. These were self-reported by participants in 2018–2020.

*Loneliness* was measured with the Three-Item Loneliness Scale, a short version of the R-UCLA Loneliness Scale^48^. It asks respondents to rate their feelings of lack of companionship, feeling left out, and feeling isolated from others on a three-point Likert scale (“often”, “some of the time,” “hardly ever or never”). These responses were summed together, resulting in a final score ranging from 3 (“not lonely”) to 9 (“very lonely”).

*Social support* was measured with the Multidimensional Scale of Perceived Social Support (MSPSS)^49^. It is a 12-item measure of perceived adequacy of social support from three sources: family, friends, and a significant other, using a 5-point Likert scale (1 = strongly disagree, 5 = strongly agree). The higher scores indicated higher perceived social support. The final score ranged from 12 to 60.

*Frequency of social contact* was measured with the question “How often do you have contact with friends or relatives that you do not live with?” assessing three forms of contact: face to face, through phone, and online. For all the three forms, respondents answered using a 5-point Likert scale (1 = daily or almost daily, 5 = never). The final score was scaled to range from 1 to 5, with higher scores indicating less frequent social contact.

*Social network heterogeneity* was measured, as in previous studies^50^, with two items (“Whom can you really count on to be dependable when you need help?” and “Whom can you really count on to care about you regardless of what is happening to you?”) from the Brief Social Support Questionnaire^51^. The questionnaire evaluated how many different types of people the respondent perceived as sources of support when needed (categories: spouse, near relative, friend, coworker, neighbour, some other person, or none). Range was from 0 to 12 sources and higher scores indicated higher heterogeneity.

Standardized scores of perceived social support, frequency of social contacts, loneliness and social network heterogeneity were used in the analyses.

#### Childhood cumulative risk factors

As in previous studies^1,52^, we examined six psychosocial domains of childhood psychosocial environment: the socioeconomic environment, the emotional environment, parental health behaviours, and stressful life events. These domains were reported by the parents using a paper-and-pencil questionnaire at the baseline examination in 1980. We assigned a binary risks status (risk vs. no risk) for each domain, which were then summed together to create an overall psychosocial risk score.

A. Socioeconomic risk cluster.

The following four components were included: parental low education (primary education), manual occupation, low income (income in the lowest 25%), and an unsteady employment history defined by the presence of any of the following: periods of unemployment, long-term sickness absence, or receipt of disability benefits. The cut-off point for the overall socioeconomic risk domain was ≥ 3.

B. Psycho-emotional risk factors.

Psycho-emotional factors were parental mental health status, childrearing style, life satisfaction, and alcohol abuse. Mental health status was in inquired as: “Have you ever been diagnosed by a health professional as having a mental health problem?” “yes” =1 point and “no” =0 points. Childrearing style and life satisfaction were measured using a 5-point scale derived from the Operation Family study, where responses indicating dissatisfaction (≥ 4) with any of the three life roles were classified as high risk. Parental heavy alcohol use was measured as the frequency of heavy drinking occasions per year and the response “once a week” indicated high risk. The cut-off point for high-risk in the psycho-emotional domain was ≥ 1.

C. Stressful life events.

Stressful life events included moving homes, change of school, divorce in the family, deaths of close relatives (parents or siblings), and hospitalization or serious illness or both of a family member. The presence of each event was assigned 1 point. The cut-off point for high risk in the stressful life events domain was ≥ 1.

D. Parental health behaviours.

Parental health behaviours included parental body mass index (BMI), level of regular physical activity, and smoking status. The following cut-off points were used to classify a high-risk level for each category: mother, father or both are obese (BMI > 30); mother, father or both are current smokers; mother, father or both indicated no regular physical activity. The cut-off point for high risk in the parental health behaviours domain was ≥ 3 risks.

E. Cumulative risk score.

The cumulative risk score was created by adding together the final scores across all the individual risk domains, which resulted in a composite score ranging from 0 to 11. This score was further dichotomized into adverse childhood environment vs. low-risk environment. Cut-off point for belonging to the high-risk group was five or more risks. A detailed description of the construction of the childhood psychosocial risk score, including information on the basis of the cut-off points and risk domain compositions has been reported previously^1^.

Table 1. Characteristics of the study participants (*N*= 1787).

**Table 1:** The associations between exposure and potential mediators (univariable logistic regression models adjusted for age and sex at baseline).

Sex	
Women	1017 (56.9%)
Men	770 (43.1%)
**Age at follow-up (years)**	
Mean (SD)	49 (± 5.0)
**Education 2011**	
Lower	1129 (76.2%)
Higher	353 (23.8%)
**Income 2011**	
Lower	399 (27.2%)
Higher	1066 (72.8%)
**Depressive symptoms 2011**	
Yes	268 (19.4%)
No	1113 (80.6%)
**Smoking status 2011**	
Non-smoker	1287 (86.2%)
Smoker	206 (13.8%)
**Excess alcohol consumption 2011**	
No	1280 (86.9%)
Yes	193 (13.1%)
**Obesity (BMI >30) 2011**	
Yes	299 (20.0%)
No	1199 (80.0%)
**Socioeconomic risks 1980**	
No	1378 (82.1%)
Yes	301 (17.9%)
**Life event risks 1980**	
No	1137 (78.6%)
Yes	310 (21.4%)
**Psycho-emotional risks 1980**	
No	1178 (67.1%)
Yes	577 (32.9%)
**Health behavior risks 1980**	
No	1221 (86.1%)
Yes	197 (13.9%)
**Cumulative psychosocial risks 1980**	
No	1014 (78.5%)
Yes	277 (21.5%)

**Table 2:** Associations between potential mediators and outcomes (univariable linear regression analyses adjusted for age, sex and childhood cumulative risk at baseline).

Exposure	OR	95% CI
	Outcome (mediator): Depressive symptoms (yes)	
Psycho-emotional risk domain	1.52	(1.15–2.01)
Life events risk domain	1.24	(0.86–1.79)
Socioeconomic risk domain	1.62	(1.15–2.26)
Health behavioural risk domain	1.23	(0.81–1.84)
**Cumulative risks**	**1.55**	**(1.07–2.21)**
	Outcome: Smoking (yes)	
Psycho-emotional risk domain	1.61	(1.18–2.18)
Life events risk domain	1.20	(0.77–1.84)
Socioeconomic risk domain	1.47	(1.00–2.12)
Health behavioural risk domain	2.38	(1.55–3.59)
**Cumulative risks**	**2.33**	**(1.57–3.43)**
	Outcome: Obesity (yes)	
Psycho-emotional risk domain	1.37	(1.04–1.78)
Life events risk domain	1.17	(0.83–1.64)
Socioeconomic risk domain	0.90	(0.63–1.26)
Health behavioural risk domain	1.49	(1.01–2.15)
**Cumulative risks**	**1.26**	**(0.89–1.78)**
	Outcome: Alcohol consumption (high)	
Psycho-emotional risk domain	1.61	(1.16–2.21)
Life events risk domain	1.19	(0.77–1.82)
Socioeconomic risk domain	0.97	(0.62–1.47)
Health behavioural risk domain	1.14	(0.69–1.83)
**Cumulative risks**	**1.27**	**(0.82–1.93)**
	Outcome: Education (low)	
Psycho-emotional risk domain	1.24	(0.95–1.60)
Life events risk domain	0.83	(0.58–1.18)
Socioeconomic risk domain	2.76	(2.03–3.74)
Health behavioural risk domain	2.16	(1.49–3.11)
**Cumulative risks**	**2.80**	**(2.01–3.90)**
	Outcome: Income (low)	
Psycho-emotional risk domain	1.11	(0.86–1.42)
Life events risk domain	1.26	(0.90–1.74)
Socioeconomic risk domain	1.72	(1.27–2.33)
Health behavioural risk domain	1.56	(1.09–2.21)
**Cumulative risks**	**1.83**	**(1.33–2.50)**

**Table 3:** The indirect effects (mediation) of depressive symptoms in the relationships between childhood and adolescence risk factors and social factors (causal mediation analyses).

Mediator	B	95% CI
	Outcome: Loneliness	
Depressive symptoms (yes)	0.69	(0.53–0.84)
Smoking status (current smoker)	0.15	(−0.03-0.34)
Obesity (BMI > 30)	0.02	(−0.13-0.17)
Alcohol consumption (high)	0.00	(−0.18-0.18)
Education (low)	−0.05	(−0.21-0.10)
Income (low)	0.27	(0.14–0.41)
	Outcome: Social support	
Depressive symptoms (yes)	−0.52	(−0.67–0.37)
Smoking status (current smoker)	−0.07	(−0.24-0.11)
Obesity (BMI > 30)	−0.05	(−0.19-0.10)
Alcohol consumption (low)	−0.12	(−0.29-0.06)
Education (low)	−0.04	(−0.18-0.11)
Income (low)	−0.29	(−0.42–0.16)
	Outcome: Frequency of contacts	
Depressive symptoms (yes)	0.11	(−0.05-0.26)
Smoking status (current smoker)	0.11	(−0.07-0.28)
Obesity (BMI > 30)	0.07	(−0.07-0.22)
Alcohol consumption. (high)	−0.04	(−0.22-0.14)
Education (low)	0.09	(−0.06-0.24)
Income (low)	0.21	(0.07–0.34)
	Outcome: Heterogeneity	
Depressive symptoms (yes)	−0.30	(−0.46–0.15)
Smoking status (current smoker)	−0.09	(−0.27-0.09)
Obesity (BMI > 30)	0.02	(−0.13-0.17)
Alcohol consumption (high)	−0.20	(−0.38–0.01)
Education (low)	−0.21	(−0.36–0.06)
Income (low)	−0.30	(−0.44–0.16)

**Table 4:** The indirect effects (mediation) of health behaviours in the relationships between childhood and adolescence risk factors and social factors (causal mediation analyses).

Outcome	Effect	Estimate	CI 95%	*p*-value	Proportion mediated
Loneliness	Direct effect	0.23	0.10–0.36	0.00	
	Indirect effect	0.05	0.01–0.09	0.01	
	Total effect	0.28	0.14–0.42	0.00	0.18
Social support	Direct effect	−0.25	−0.37-−0.13	0.00	
	Indirect effect	−0.05	−0.08-−0.02	0.00	
	Total effect	−0.30	−0.41-−0.18	0.00	0.17
Frequency of contact	Direct effect	0.16	0.03–0.28	0.02	
	Indirect effect	0.01	−0.00-0.02	0.20	
	Total effect	0.16	0.04–0.29	0.01	0.05
Heterogeneity	Direct effect	−0.34	−0.44-−0.22	0.00	
	Indirect effect	−0.03	−0.05-−0.00	0.03	
	Total effect	−0.36	−0.48-−0.25	0.00	0.07

**Table 5:** The indirect effects (mediation) of socioeconomic factors in the relationships between childhood and adolescence risk factors and social factors (causal mediation analyses).

outcome name	Mediator	Effect	Estimate	CI 95%	*p*-value	Proportion mediated
Loneliness	Smoking status	Direct effect	0.32	0.21–0.43	0.00	
		Indirect effect	0.01	−0.01-0.03	0.49	
		Total effect	0.33	0.22–0.44	0.00	0.02
Social support	Smoking status	Direct effect	−0.30	−0.43–0.18	0.00	
		Indirect effect	−0.00	−0.02-0.01	0.67	
		Total effect	−0.30	−0.43–0.18	0.00	0.01
Frequency of contact	Smoking status	Direct effect	0.16	0.02–0.29	0.02	
		Indirect effect	0.01	−0.01-0.02	0.32	
		Total effect	0.17	0.03–0.30	0.01	0.04
Heterogeneity	Smoking status	Direct effect	−0.35	−0.46–0.24	0.00	
		Indirect effect	−0.00	−0.02-0.01	0.63	
		Total effect	−0.35	−0.46–0.25	0.00	0.01
Loneliness	Obesity	Direct effect	0.30	0.18–0.42	0.00	
		Indirect effect	0.00	−0.01-0.01	0.55	
		Total effect	0.30	0.18–0.42	0.00	0.01
Social support	Obesity	Direct effect	−0.33	−0.44–0.22	0.00	
		Indirect effect	−0.00	−0.01-0.00	0.68	
		Total effect	−0.33	−0.44–0.22	0.00	0.00
Frequency of contact	Obesity	Direct effect	0.16	0.04–0.27	0.01	
		Indirect effect	0.00	−0.00-0.00	0.83	
		Total effect	0.16	0.04–0.27	0.01	0.00
Heterogeneity	Obesity	Direct effect	−0.36	−0.49–0.24	0.00	
		Indirect effect	−0.00	−0.00-0.00	0.89	
		Total effect	−0.36	−0.49–0.24	0.00	0.00
Loneliness	Alcohol consumption	Direct effect	0.29	0.17–0.42	0.00	
		Indirect effect	0.00	−0.00-0.01	0.85	
		Total effect	0.29	0.17–0.42	0.00	0.00
Social support	Alcohol consumption	Direct effect	−0.29	−0.40–0.17	0.00	
		Indirect effect	−0.00	−0.01-0.00	0.26	
		Total effect	−0.29	−0.40–0.18	0.00	0.01
Frequency of contact	Alcohol consumption	Direct effect	0.18	0.05–0.31	0.01	
		Indirect effect	−0.00	−0.01-0.00	0.45	
		Total effect	0.18	0.05–0.31	0.01	−0.01
Heterogeneity	Alcohol consumption	Direct effect	−0.34	−0.46–0.23	0.00	
		Indirect effect	−0.00	−0.01-0.00	0.28	
		Total effect	−0.35	−0.46–0.23	0.00	0.01

**Table 6:** The indirect effects (mediation) of socioeconomic factors in the relationships between childhood and adolescence risk factors and social factors (causal mediation analyses).

outcome name	Mediators	Effect	Estimate	CI 95%	*p*-value	Proportion mediated
Loneliness	Education	Direct effect	0.31	0.20–0.43	0.00	
		Indirect effect	−0.00	−0.04-0.03	0.86	
		Total effect	0.31	0.20–0.42	0.00	−0.01
Social support	Education	Direct effect	−0.30	−0.43–0.17	0.00	
		Indirect effect	−0.01	−0.03-0.02	0.71	
		Total effect	−0.30	−0.43–0.18	0.00	0.02
Frequency of contact	Education	Direct effect	0.17	0.04–0.30	0.01	
		Indirect effect	−0.00	−0.03-0.02	0.84	
		Total effect	0.17	0.04–0.30	0.01	−0.02
Heterogeneity	Education	Direct effect	−0.33	−0.45–0.21	0.00	
		Indirect effect	−0.03	−0.06–0.00	0.04	
		Total effect	−0.36	−0.47–0.24	0.00	0.08
Loneliness	Income	Direct effect	0.28	0.17–0.40	0.00	
		Indirect effect	0.03	0.01–0.05	0.00	
		Total effect	0.31	0.20–0.42	0.00	0.09
Social support	Income	Direct effect	−0.28	−0.40–0.16	0.00	
		Indirect effect	−0.03	−0.06–0.01	0.00	
		Total effect	−0.32	−0.44–0.20	0.00	0.11
Frequency of contact	Income	Direct effect	0.16	0.02–0.30	0.02	
		Indirect effect	0.01	−0.00-0.03	0.07	
		Total effect	0.17	0.04–0.31	0.01	0.09
Heterogeneity	Income	Direct effect	−0.33	−0.44–0.22	0.00	
		Indirect effect	−0.03	−0.06–0.01	0.00	
		Total effect	−0.36	−0.47–0.25	0.00	0.09

#### Potential confounders and mediators

Potential mediators included dichotomous (0/1) measures of (A) depressive symptoms, (B) socioeconomic status (SES) and (C) health behaviours recorded in adulthood (2011). Depressive symptoms in 2011 were assessed using the Beck Depression Inventory (BDI)^53,54^ where subjects were asked to respond to 21 items with one of four alternative response options ranging from minimal to severe symptoms of depression. The BDI sum score was dichotomized using a cut-off point of 9 or more to identify those in the highest 20% of the BDI distribution (high risk). SES was measured by personal gross income and total years of education. Participant’s gross annual income reported by the participant on an eight-point scale (< 10,000€, 10,001–20,000€, 20,001–30,000€, 30,001–40,000€, 40,001–50,000€, 50,001–60,000€, 60,001–70,000€, > 70,000€) in 2011. For the analysis, the income measure was dichotomized with participants in the lowest 25% classified as “low income” group (high risk). Adult educational attainment was measured as the highest level of completed or attended education in 2011 and categorized as *low*=primary and lower secondary education, *intermediate*=upper secondary education, and *academic*=polytechnic or university degree. This variable was then dichotomized, with low and intermediate education classified as “low educational attainment” (high risk). Health behaviours included alcohol consumption, BMI and smoking status. Alcohol consumption was the self-reported frequency of using ≥ 6 units of alcohol beverages ranging from 1 (two times a week or more often) to 6 (never). High alcohol consumption was defined as consuming ≥ 6 units more often than once a month (high risk). The participants’ BMI was calculated as weight (kg)/height (m^2^). Weight was measured in light clothing without shoes with a Seca digital weight scale with an accuracy of 0.1 kg. Height was measured with a Seca anthropometer using a wall-mounted stadiometer accurate to 0.1 cm, as described in detail previously^47^. Obesity (BMI > 30 kg/m^2^) (high risk) was calculated based on height and weight and was used in the main analyses^55^. Smoking status was assessed by a questionnaire and classified as current smokers (high risk) vs. previous or non-smokers. We chose the measurement point between the baseline and the outcome that had all the measures needed for the analyses (in 2011). In that way we thought we had the most consistent design for the study, because some of the confounders were only measured in adulthood.

#### Statistical analyses

The associations between the childhood and adolescence psychosocial risks (exposure measured in 1980), mediators (depressive symptoms, SES and health behaviours measured in 2011), and outcomes (loneliness, perceived social support, frequency of social contact and social network heterogeneity measured in 2018–2020) were first analysed using linear regression models. To explore the prerequisites for the mediation analysis, we also assessed exposure-outcome and mediator-outcome associations using linear regression. For the exposure-mediator associations we applied logistic regression. All regression models were adjusted for participant’s age and sex at baseline and the exposure-mediator associations additionally for cumulative childhood risks.

To examine the mediating role of depressive symptoms, SES, and health behaviours in the associations between childhood psychosocial risks and social relations in midlife, we used causal mediation analysis^56^. This analysis decomposes the total association of childhood or adolescence psychosocial risk factors on adult social relations into a direct association and an indirect association (i.e., association mediated through depressive symptoms, SES or health behaviours in adulthood). In this framework, we used closed-form parameter function estimation and delta method inference^57,58^.

All analyses were conducted in R (version 4.3.2) and the causal mediation analysis was conducted using the “CMAverse” (for causal mediation analysis)^36^ package.

## Supplementary Information

Below is the link to the electronic supplementary material.

Supplementary Material 1

## Data Availability

Data used in the current study may be obtained from the University of Turku. Restrictions apply to the availability of these data, which were used under licence for this study. For information on accessing the data see: https://youngfinnsstudy.utu.fi.

## Declarations

### Manuscript statistics

Words in Abstract 266, in text 4015, 4 tables, 1 Figure.
