# Supplementary material for: The associations of childhood and adolescence psychosocial risks with social relations in midlife
Source: Sci Rep. 2026 Jul 1;16:30275. doi: 10.1038/s41598-026-58873-5 (PMC13623642; doi:10.1038/s41598-026-58873-5)
Supplement: Supplementary file 1 [file 41598_2026_58873_MOESM1_ESM.docx]

Supplement for Elovainio, M., Komulainen, K., Pahkala, K., Rovio, S., Lehtimäki, T., Viikari, J., Määttänen, I, Raitakari, OT., & Pulkki – Råback, L. **Childhood and adolescence psychosocial risks and social relations in midlife: evidence from 38 -year follow-up study**

Supplement figure 1. Flow chart of study sample selection

Supplement Figure 2. Association between childhood/ adolescence psychosocial risk domains and four indicators of social relations in adulthood (linear regression analyses), adjusted for age and sex.

| Stable 1. Process chart of the construction of the psychosocial risks | | | |
| --- | --- | --- | --- |
| Components | Definition of risk level | Absent | Present |
| Socioeconomic risks | | | |
| Occupational status | Blue collar | 0 | 1 |
| Educational level | Comprehensive aducation | 0 | 1 |
| Family income | Annual income in lowest quartile | 0 | 1 |
| Occupational stability | Unsteady employment (periods of unemployment, long-term sickness absence, or receipt of disability benefits | 0 | 1 |
| Psycho-emotional risk factors | | | |
| Poor parental mental health | Diagnosis for mental disorder | 0 | 1 |
| Poor parental nurturance | Negative to the nurturance scale | 0 | 1 |
| Poor parental life satisfaction | Negative reply to the satisfaction scale | 0 | 1 |
| Heavy alcohol use | Intoxication >3 times a year | 0 | 1 |
| Parental health behaviours | | | |
| Energy intake (mother) | Body mass index ≥30.0 | 0 | 1 |
| Energy intake (father) | Body mass index ≥30.0 | 0 | 1 |
| Smoking (mother) | Daily smoking | 0 | 1 |
| Smoking (father) | Daily smoking | 0 | 1 |
| Physical activity (mother) | Exercise <1 times per week | 0 | 1 |
| Physical activity (father) | Exercise <1 times per week | 0 | 1 |
| Stressful life events | | | |
| Stability of living environment | Change of residence during youth | 0 | 1 |
| Stability of school environment | Change of school during youth | 0 | 1 |
| Stability of family environment | Parental divorce or separation | 0 | 1 |
| Loss of significant persons | Death of family member | 0 | 1 |
| Health-related events | Long-term hospitalization/disease | 0 | 1 |

Supplement Figure 3. Means and standard deviations of social relations variables
